# Increased Resistance to Biotrophic Pathogens in the Arabidopsis *Constitutive Induced Resistance 1* Mutant Is EDS1 and PAD4-Dependent and Modulated by Environmental Temperature

**DOI:** 10.1371/journal.pone.0109853

**Published:** 2014-10-10

**Authors:** Maryke Carstens, Tyronne K. McCrindle, Nicolette Adams, Anastashia Diener, Delroy T. Guzha, Shane L. Murray, Jane E. Parker, Katherine J. Denby, Robert A. Ingle

**Affiliations:** 1 Department of Molecular and Cell Biology, University of Cape Town, Rondebosch, South Africa; 2 Department of Plant Microbe Interactions, Max Planck Institute for Plant Breeding Research, Köln, Germany; 3 School of Life Sciences and Warwick Systems Biology Centre, University of Warwick, Coventry, United Kingdom; Virginia Tech, United States of America

## Abstract

The Arabidopsis *constitutive induced resistance 1* (*cir1*) mutant displays salicylic acid (SA)-dependent constitutive expression of defence genes and enhanced resistance to biotrophic pathogens. To further characterise the role of CIR1 in plant immunity we conducted epistasis analyses with two key components of the SA-signalling branch of the defence network, ENHANCED DISEASE SUSCEPTIBILITY1 (EDS1) and PHYTOALEXIN DEFICIENT4 (PAD4). We demonstrate that the constitutive defence phenotypes of *cir1* require both EDS1 and PAD4, indicating that CIR1 lies upstream of the EDS1-PAD4 regulatory node in the immune signalling network. In light of this finding we examined EDS1 expression in *cir1* and observed increased protein, but not mRNA levels in this mutant, suggesting that CIR1 might act as a negative regulator of EDS1 via a post-transcriptional mechanism. Finally, as environmental temperature is known to influence the outcome of plant-pathogen interactions, we analysed *cir1* plants grown at 18, 22 or 25°C. We found that susceptibility to *Pseudomonas syringae* pv. *tomato* (*Pst*) DC3000 is modulated by temperature in *cir1*. Greatest resistance to this pathogen (relative to *PR-1:LUC* control plants) was observed at 18°C, while at 25°C no difference in susceptibility between *cir1* and control plants was apparent. The increase in resistance to *Pst* DC3000 at 18°C correlated with a stunted growth phenotype, suggesting that activation of defence responses may be enhanced at lower temperatures in the *cir1* mutant.

## Introduction

Plants have a robust innate immune system that affords protection against attack by potential pathogens in their local environment. Detection of pathogen associated molecular patterns (PAMPs) such as flagellin by pattern recognition receptors at the plasma membrane leads to activation of PAMP-triggered immunity (PTI) [1], [2]. Successful phytopathogens have evolved mechanisms, including effectors, to subvert or suppress PTI, allowing them to successfully colonise the plant host [1], [3]. This in turn led to the evolution of effector-triggered immunity (ETI) in plants, which relies on the direct or indirect detection of pathogen effectors by cognate host resistance (R) proteins [1], [4]. While there is a significant overlap between these two branches of the innate immune system, ETI is generally regarded as a stronger and more rapid response, and is associated with the hypersensitive (HR) response [1], [3]. The final layer of innate immunity is systemic acquired resistance (SAR), whereby infection of one part of a plant leads to increased resistance of uninfected tissues to subsequent pathogen challenge [4]. SAR is thought to be established by co-ordinated expression of an array of anti-microbial *pathogenesis related* (*PR*) genes [4], [5]. All three branches of innate immunity rely on large scale transcriptional re-programming of the host plant, activated via a complex network that is influenced by the crosstalk between salicylic acid (SA), jasmonic acid (JA) and ethylene (Et) signalling [6], [7].

Two key signalling components in the SA-signalling branch of the defence network are ENHANCED DISEASE SUSCEPTIBILITY1 (EDS1) and PHYTOALEXIN DEFICIENT4 (PAD4) [8], [9], both of which show homology to eukaryotic acyl lipases. The EDS1-PAD4 node has long been recognised as a central regulator of PTI and of Toll–interleukin-1 receptor–nucleotide binding–leucine-rich repeat (TIR-NB-LRR) R protein mediated ETI against biotrophic and hemi-biotrophic pathogens [9]–[11]. Recent evidence suggests that EDS1 may also play a role in ETI mediated via coiled coil-NB-LRR R proteins, as EDS1 and SA accumulation have been shown to function redundantly in RPS2 and RPP8-mediated resistance against avirulent pathogens [12]. Both EDS1 and PAD4 are required for SA accumulation in response to *Pseudomonas syringe* pv. *tomato* (*Pst*) DC3000 or *Pst* DC3000 *avrRps4* [13], and *EDS1* and *PAD4* gene expression is SA-inducible, suggesting the existing of a positive feedback loop [11], [13].

Protein-protein interaction studies have identified the presence of EDS1 homodimers, as well as EDS1-PAD4, EDS1-SAG101 and EDS1-PAD4-SAG101 protein complexes in plant cells [13]–[16]. The formation of the EDS1-PAD4 complex is required for PTI against virulent pathogens, full accumulation of SA and the establishment of SAR, but not for TIR-NB-LRR mediated ETI [15]. While EDS1 homodimers are present predominantly in the cytoplasm, the EDS1-PAD4 complex is found in the cytoplasm and nucleus, and it has been suggested that nuclear EDS1 acts as a transcriptional regulator [14], [15]. Enhanced export of EDS1 from the nucleus was found to increase susceptibility to both virulent and avirulent *Pst* DC3000, as well as *Hyaloperonospora arabidopsidis* Emwa1 [17], but co-ordination of cytoplasmic and nuclear EDS1 levels may also be important in the plant immune response [17]. In line with its central role in innate immunity in Arabidopsis, EDS1 is targeted by the *Pst* effectors AvrRps4 and HopA1, and in accordance with the guard hypothesis of Van der Biezen and Jones [18], EDS1 is found in association with the cognate TIR-NB-LRR R proteins RPS4 and RPS6 [19].

EDS1 and PAD4 were identified in Arabidopsis by screening for altered susceptibility to pathogen challenge, and mutant screens have been widely used to dissect the defence signalling network. One class of gain-of-resistance mutants that display SAR-like constitutive disease resistance are the *constitutive expressor of PR genes* (*cpr*) mutants [20]–[22]. These mutants display SA signalling-dependent constitutive expression of *PR* genes and enhanced resistance to virulent biotrophic pathogens [21]. The *cpr*-type mutants can broadly be divided into two groups [23], those that display constitutive HR-like cell death such as *cpr5* and *lsd1*, and those that do not, including *cpr1* and *dnd1*. The *cir1* (*constitutively induced resistance 1*) mutant belongs to the second class of *cpr* mutants, and was identified in a mutant screen for increased luciferase activity in Col-0 plants carrying a *PR-1:LUC* reporter [24]. The *cir1* mutation is recessive, and homozygous *cir1* plants display increased resistance to virulent *Pst* DC3000 and *H. arabidopsidis* and constitutive expression of SA-dependent defence genes such as *PR-1, PR-5* and *WRKY53* (as well as the JA/Et-dependent *PDF1.2*) in the absence of pathogen challenge [24], [25]. As reported for other *cpr*-type mutants, SA accumulation is essential for the increased resistance to virulent biotrophic pathogens displayed by *cir1*, which appears to be mediated by both NPR1-dependent and independent signalling pathways, since *cir1 npr1* double mutants displayed only partial suppression of *cir1*-mediated resistance [24]. Although *cir1* displays increased *PDF1.2* expression, it does not display increased resistance to the necrotrophic fungal pathogen *Botrytis cinerea* [23]. The *CIR1* gene maps to the lower arm of chromosome IV and complementation tests have revealed that it is not allelic to previously reported *cpr* mutations in this region including *cpr1* [24].

Epistasis analyses have revealed that EDS1 and PAD4 are required for constitutive *PR* expression and enhanced disease resistance in several *cpr* mutants including *cpr1* and *cpr6* [21], [26]. Given these results and the pivotal role of the EDS1-PAD4 regulatory node in SA-mediated defence against biotrophic pathogens we investigated whether *cir1*-mediated resistance to *Pst* DC3000 and *H. arabidopsidis* also requires EDS1 and/or PAD4, and whether CIR1 might in turn regulate EDS1 expression. Our data indicate that CIR1 is a negative regulator of innate immunity that lies upstream of EDS1 and PAD4 in the defence signalling network, and suggest that CIR1 may be involved in the post-transcriptional regulation of EDS1. In addition, we show that the defence and growth phenotypes of the *cir1* mutant are modulated by environmental temperature.

## Results

### *cir1*-mediated resistance to *Pst* DC3000 and *H. arabidopsidis* requires EDS1 and PAD4

The increased resistance to *Pst* DC3000 and *H. arabidopsidis* Noco2 displayed by the *cir1* mutant has previously been shown to be SA-dependent [24]. EDS1 and PAD4 are two key players in defence against *Pst* DC3000 and *H. arabidopsidis* and are essential for SA accumulation in response to infection by these pathogens [13]. To determine whether *cir1*-mediated resistance to these pathogens is also dependent on EDS1 and PAD4, we generated *cir1 eds1* and *cir1 pad4* double mutants and examined their disease susceptibility profiles.

**Figure 1:**
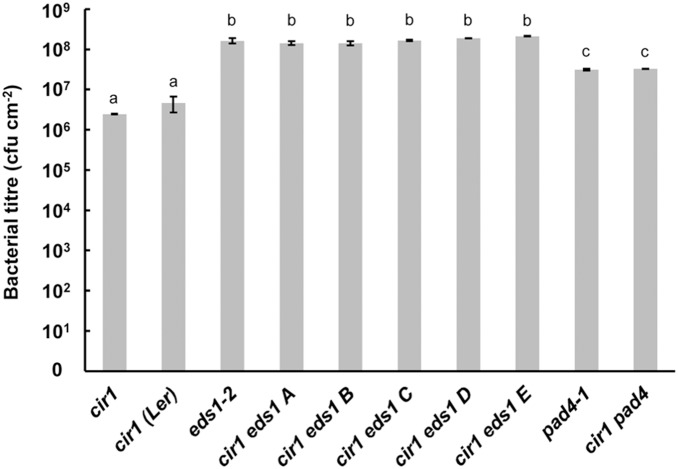
EDS1 and PAD4 are required for *cir1*-mediated resistance to *Pst* DC3000. Four-week old plants grown at 22°C were pressure inoculated with *Pst* DC3000 (10^6^ cfu mL^−1^) and bacterial titres determined at 48 hpi. Data shown are mean values ± SEM (n = 3) from one experiment representative of three independent experiments. Mean bacterial titres (cfu cm^−2^) with different letters are significantly different (*p*<0.05).

As *eds1* is in the Ler background, while *cir1* is in the Col-0 background, we analysed five independently generated *cir1 eds1* double mutants to control for any effects of a mixed Col-0/Ler background on resistance to *Pst* DC3000. Bacterial titres in these five lines at 48 h post-infection (hpi) were not significantly different from those observed in the *eds1-2* mutant, while those observed in *cir1* were significantly lower (Figure 1). There was no significant difference in bacterial titres between the *cir1* mutant in the Col-0 background (*cir1*) and *cir1* plants generated by crossing the single mutant to wild-type Ler plants (*cir1* Ler), indicating that a mixed Col-0/Ler genetic background has no effect on *cir1*-mediated resistance to *Pst* DC3000 (Figure 1). Similar results were observed for PAD4; *cir1* displayed significantly lower bacterial titres 48 hpi than *pad4-1*, while no statistically significant difference in bacterial titres was observed between *pad4-1* and *cir1 pad4* plants (Figure 1). Thus, both EDS1 and PAD4 are required for *cir1*-mediated resistance to *Pst* DC3000, and are epistatic to CIR1.

**Figure 2:**
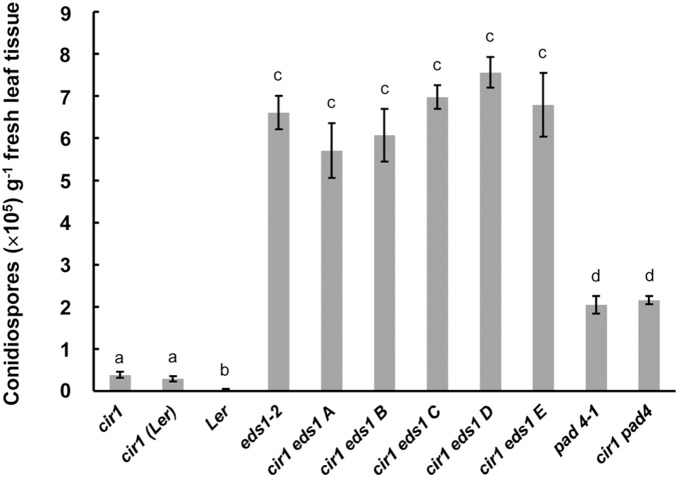
EDS1 and PAD4 are required for *cir1*-mediated resistance to *H. arabidopsidis* Noco2. Four-week old plants grown at 22°C were infected with *H. arabidopsidis* (10^4^ conidiospores mL^−1^) and condiospore load determined at 7 dpi. ANOVA revealed a significant effect of host genotype (*p*<0.001) on conidiospore load. Mean conidiospore counts (spores g^−1^ fresh weight) with different letters are significantly different (*p*<0.05). Data shown are mean values ± SEM (n = 4) from one experiment representative of three independent experiments.

We also examined the resistance profile of the double mutants to *H. arabidopsidis* Noco2. This pathogen is virulent on the Arabidopsis Col-0 ecotype, but avirulent on Ler due to the presence of the *RPP5* resistance gene [27]. EDS1 is required for RPP5-mediated resistance to *H. arabidopsidis* [9], as reflected in the significantly higher conidiospore production and mycelial growth observed in *eds1-2* compared to Ler (Figure 2 and S1). Conidiospore production in the five independently derived *cir1 eds1* lines was not significantly different from that observed in *eds1-2*, while that in *cir1* was significantly lower (Figure 2 and S1). Again, similar results were observed with the *pad4* mutants, with no statistically significant difference in conidiospore production between *pad4-1* and *cir1 pad4*. However, disease symptoms were less severe in *pad4-1* in comparison to *eds1-2* as previously reported [15]. Together these data suggest that EDS1 and PAD4 are also required for *cir1*-mediated resistance to *H. arabidopsidis*, and again function downstream of CIR1. We observed that *cir1* in the Ler (*cir1* Ler) background displayed the reduced susceptibility to *H. arabidopsidis* observed in *cir1* in the Col-0 backround, rather than the total resistance displayed by wild-type Ler plants (Figure 2). Intermediate susceptibility to this pathogen has previously been reported in Col-0×Ler crosses [27].

### EDS1 and PAD4 are required for *cir1*-modulated defence gene expression

**Figure 3:**
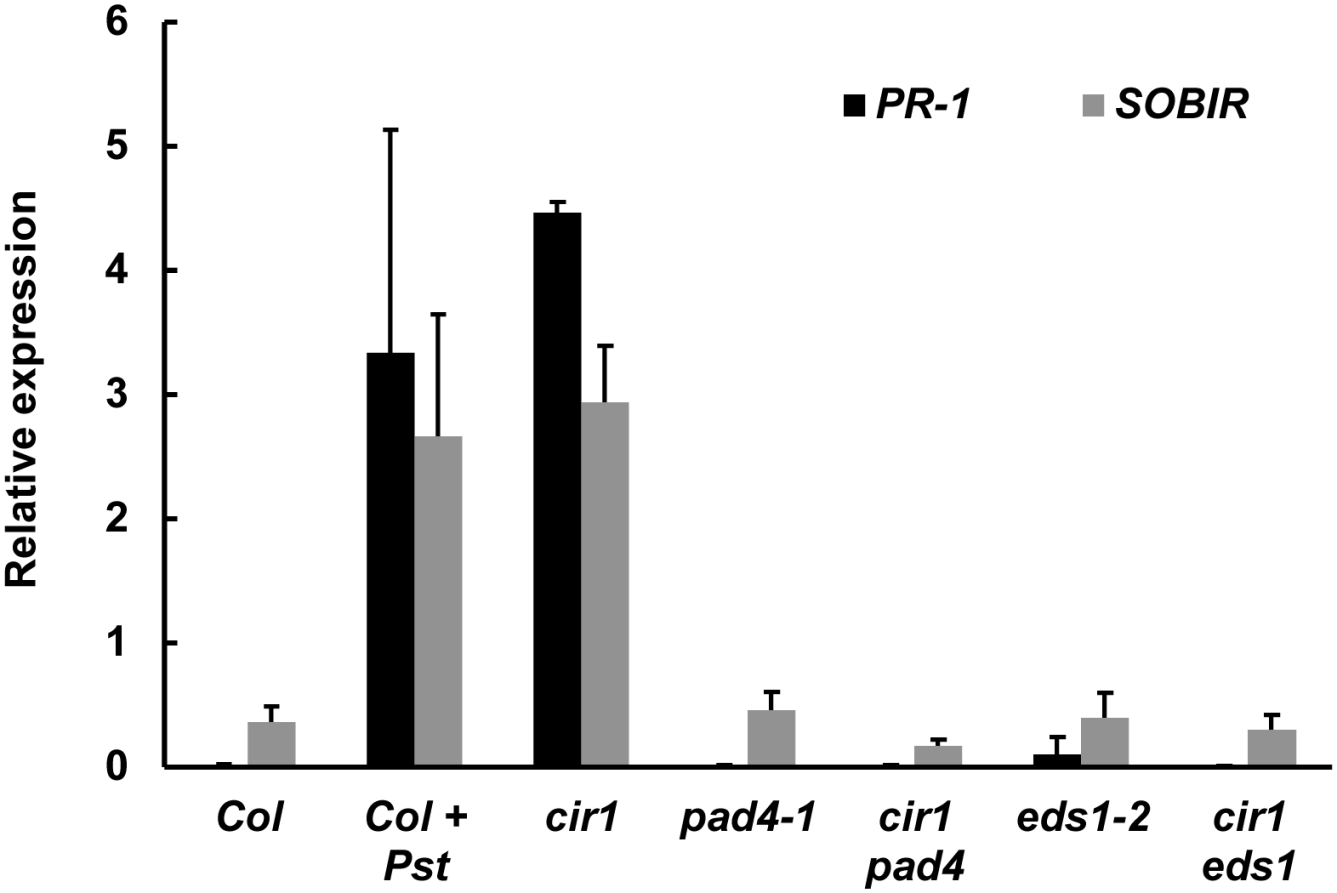
EDS1 and PAD4 are required for *cir1*-mediated constitutive defence gene expression. Relative expression values for At2g14160 (*PR-1*) and At2g31880 (*suppressor of BIR1*) were determined in four-week-old plants grown at 22°C using qPCR, with normalisation to *Actin2* expression. Col-0 + Pst plants were inoculated with *Pst* DC3000 (10^6^ cfu mL^−1^) and tissue harvested after 24 h. Values shown are the means of two independent biological repeats + SD.

As both EDS1 and PAD4 are required for *cir1*-mediated resistance against virulent biotrophic pathogens, we examined the expression of two downstream defence genes that are SA responsive and require EDS1 and PAD4 for up-regulation in response to *Pst* DC3000 infection [28], [29]. Quantitative PCR analysis confirmed that At2g14160 (*PR-1*) and At2g31880 (*suppressor of BAK1-interacting receptor-like kinase 1, SOBIR1*) were up-regulated in Col-0 plants 24 hpi with *Pst* DC3000, and that mRNA levels were elevated in uninfected *cir1* plants compared to uninfected Col-0 plants (Figure 3). However, the *cir1 eds1* and *cir1 pad4* double mutants again phenocopied the single *eds1-2* or *pad4-1* mutants rather than *cir1*, as mRNA levels of both genes were not elevated in these plants (Figure 3). These data indicate that EDS1 and PAD4 are required for elevated constitutive expression of these defence genes in *cir1*, and support the hypothesis that CIR1 is upstream of the EDS1-PAD4 regulatory node.

### EDS1 expression is constitutively higher in the *cir1* mutant

**Figure 4:**
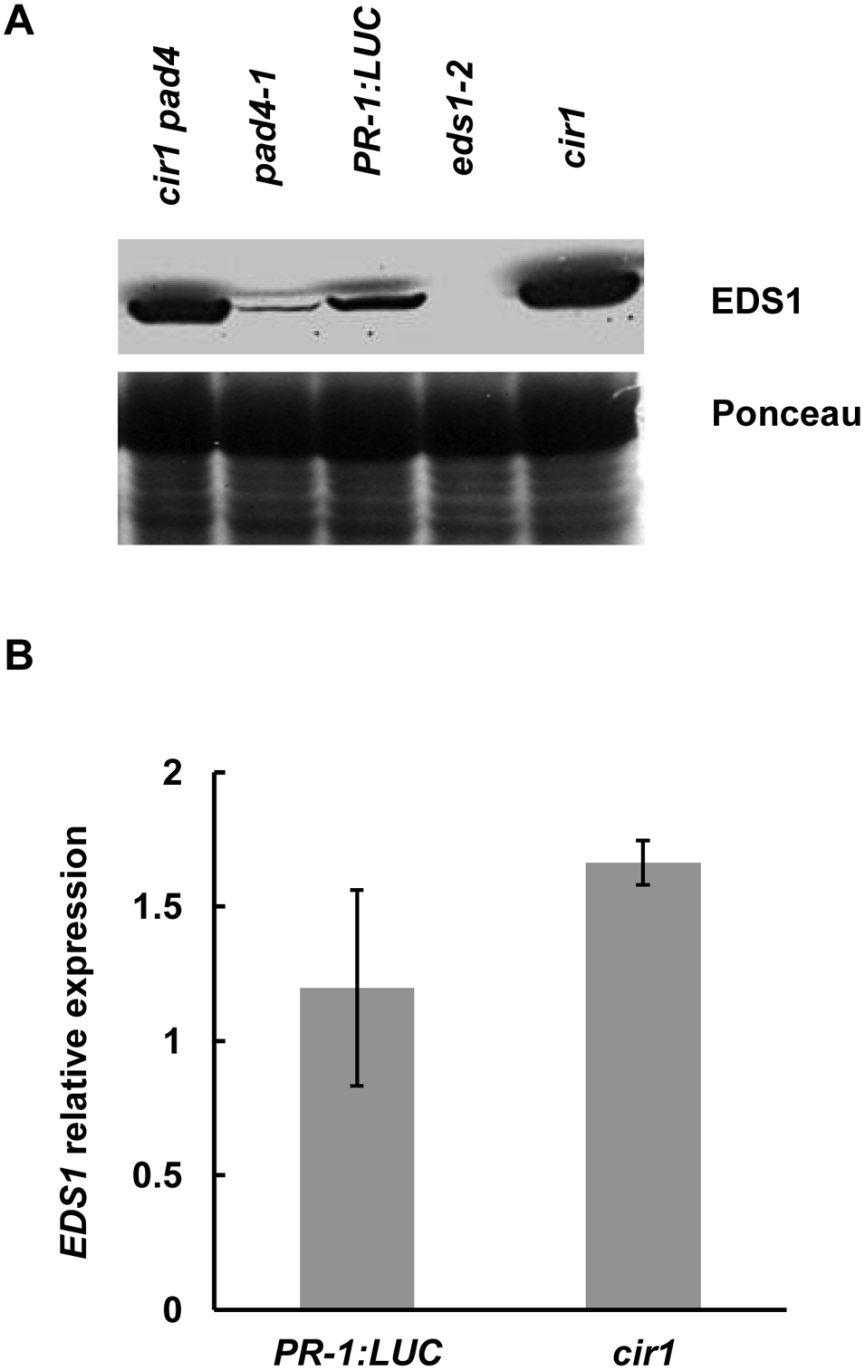
EDS1 protein but not mRNA levels are constitutively higher in the *cir1* mutant. (A) Total protein from 4-week-old plants grown at 22°C was separated by SDS–PAGE, transferred to nitrocellulose membrane and probed with an EDS1 antibody. Equal loading of the gel was verified by Ponceau staining of the membrane after protein transfer. This experiment was repeated twice with the same results. (B) Relative *EDS1* expression in 4-week-old *cir1* and *PR-1:LUC* plants was determined using qPCR, with normalization to *Actin2* expression levels. Each value is the mean of three independent biological repeats ± SEM. This experiment was repeated three times with the same results.

Given that CIR1 functions upstream of EDS1 and PAD4, we next examined whether CIR1 might regulate EDS1 expression in Arabidopsis. Western blot analysis revealed that EDS1 proteins levels were constitutively higher in uninfected *cir1* plants in comparison to *PR-1:LUC* control plants (the genetic background for the *cir1* mutant), and also in *cir1 pad4* plants versus the *pad4-1* single mutant (Figure 4A). However, analysis of *EDS1* steady-state transcript levels using qPCR revealed no statistically significant difference between *cir1* and *PR-1:LUC* control plants (Figure 4B), suggesting that CIR1 may exert its regulatory effect on EDS1 via a post-transcriptional mechanism.

### The *cir1* growth and disease resistance phenotypes are temperature-sensitive

**Figure 5:**
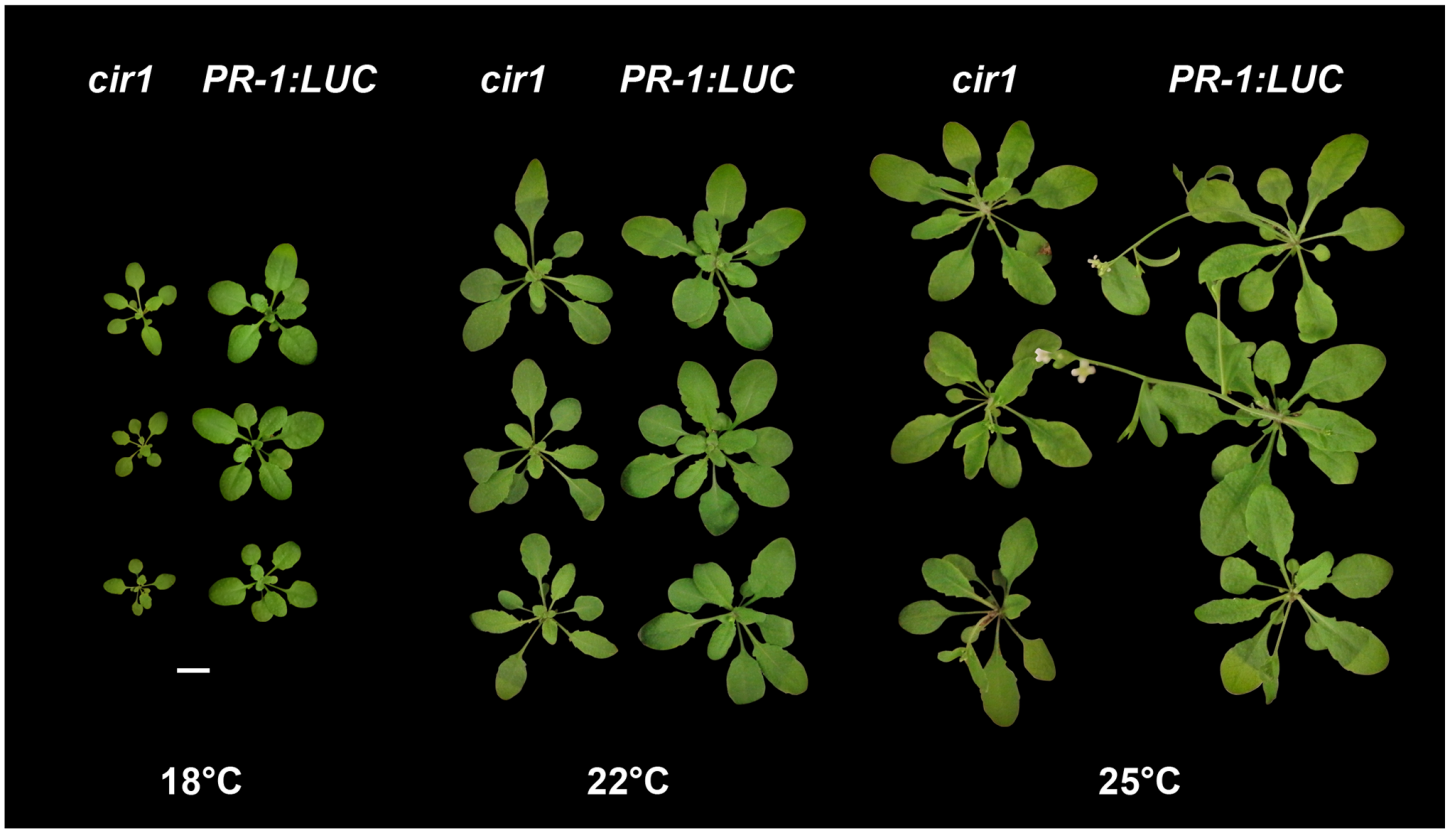
The *cir1* mutant displays a temperature-sensitive growth phenotype. Representative *cir1* and *PR-1:LUC* plants grown for four weeks under a 16 h light/8 h dark cycle at 18, 22 or 25°C are shown. Scale bar indicates a distance of 10 mm.

**Figure 6:**
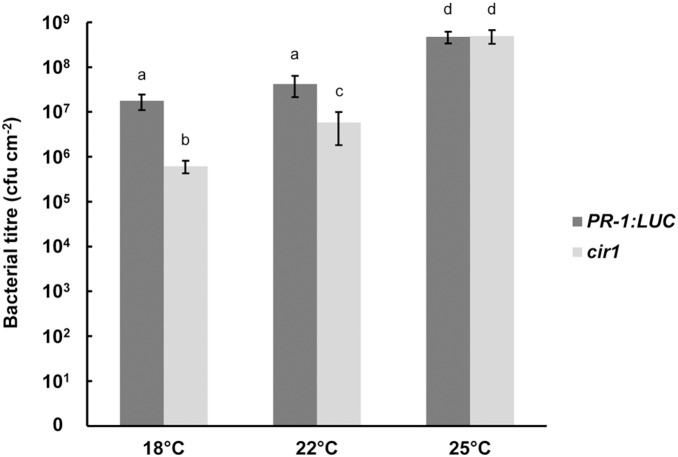
Susceptibility to *Pst* DC3000 is modulated by temperature in *cir1*. Four-week-old *cir1* and *PR-1:LUC* plants grown at 18, 22 or 25°C were pressure inoculated with *Pst* DC3000 (10^6^ cfu mL-1) and bacterial titres determined at 48 hpi. Data shown are mean values ± SEM (n = 8–10). ANOVA revealed a significant effect of host genotype (*p*<0.001) and temperature (*p*<0.001) on bacterial titres at 48 hpi, A significant interaction between these two variables (*p* = 0.015) indicates that they combine non-additively to influence bacterial growth. Mean bacterial titres (cfu cm^−2^) with different letters are significantly different (*p*<0.05).

**Figure 7:**
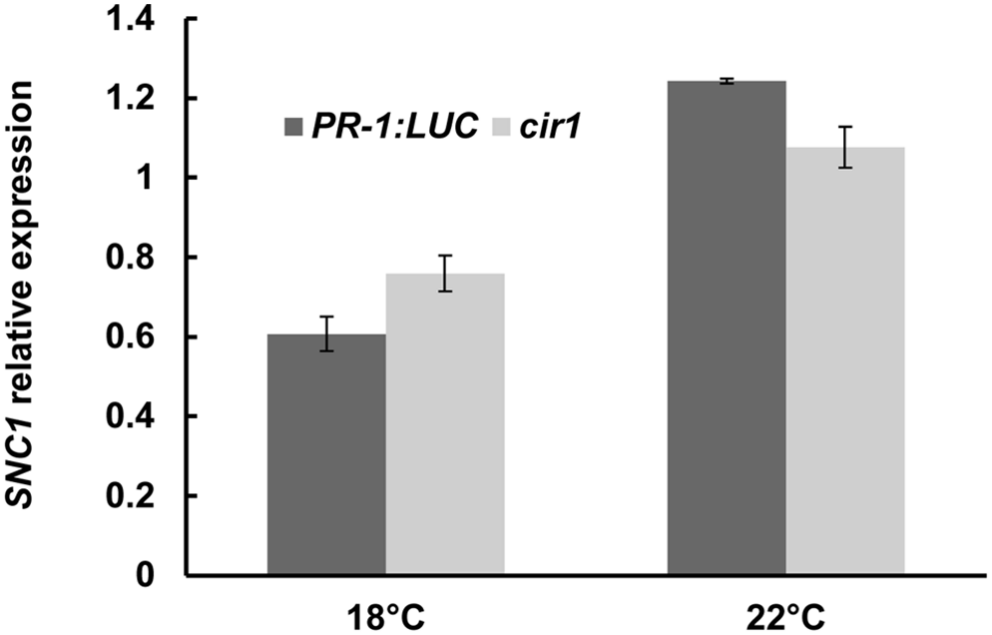
*SNC1* transcript levels are not elevated in *cir1*. Relative *SNC1* expression in 4-week-old *cir1* and *PR-1:LUC* plants grown at 18 or 22°C was determined using qPCR, with normalization to *Actin2* expression levels. Each value is the mean of three independent biological repeats ± SEM.

A number of gain-of-resistance mutants display a stunted growth phenotype, which is thought to result from the fitness cost of constitutive activation of immune responses [30]. In several mutants, including *cpr1*, *bonzai1* (*bon1*) and *suppressor of npr1-1, constitutive 1* (*snc1*), this phenotype is temperature dependent, manifesting only at lower growth temperatures [22], [31]. While *cir1* does not exhibit the dwarf stature characteristic of these mutants at 22°C (our standard growth temperature for Arabidopsis), it does display moderately reduced stature in comparison to *PR-1:LUC* control plants (Figure 5). We thus examined the growth phenotype and resistance to *Pst* DC3000 of the *cir1* mutant when grown at 18, 22 or 25°C. We observed that *cir1* plants displayed greatly reduced stature compared to *PR-1:LUC* control plants at 18°C, while at 25°C no obvious difference in size was evident (Figure 5). ANOVA of *Pst* DC3000 titres 48 hpi revealed significant (*p*<0.001) effects of both temperature and genotype on resistance to *Pst* DC3000, and a significant interaction term (genotype*temperature, *p* = 0.015), indicating that the effect of genotype on resistance to *Pst* DC3000 is modulated by environmental temperature. While bacterial titres were on average 8.1-fold (± 2.3) lower in *cir1* plants compared to *PR-1:LUC* plants when grown at 22°C, at 18°C bacterial titres in the *cir1* mutant were on average 30.1-fold (±8.1) lower than those in control plants (Figure 6). In contrast, when grown at 25°C there was no significant difference in bacterial titres 48 hpi (Figure 6), suggesting that enhanced resistance to *Pst* DC3000 was abolished in the *cir1* mutant at this temperature. The auto-immune phenotypes displayed by several *cpr*-type mutants including *cpr1* and *bon1* when grown at 22°C have been linked to increased expression of the TIR-NB-LRR protein SNC1, with increased *SNC1* mRNA levels reported in *cpr1* and *bon1* [31], [37]. However, qPCR analysis revealed that *SNC1* transcript levels were not significantly higher in *cir1* versus *PR-1:LUC* plants when grown at either 18 or 22°C (Figure 7).

## Discussion

The EDS1-PAD4 regulatory node plays a critical role in both PTI and ETI against biotrophic pathogens in Arabidopsis. Here we investigated whether the enhanced resistance to *Pst* DC3000 and *H. arabidopsidis* and constitutive expression of SA-dependent defence genes displayed by the *cir1* mutant requires EDS1 and PAD4 by generating double mutants. We observed that both *cir1 eds1* and *cir1 pad4* plants displayed the enhanced susceptibility to *Pst* DC3000 and *H. arabidopsidis* that is characteristic of the single *eds1-2* and *pad4-1* null mutants (Figures 1 & 2). Similarly, analysis of steady state levels of two SA-dependent defence genes revealed that their elevated expression in uninfected *cir1* plants requires both EDS1 and PAD4 (Figure 3). These results are in line with those previously observed for a number of other gain-of-resistance mutants including *cpr1*, *cpr6*, *bon1*, *snc1* and *suppressor of rps4-RLD 1* (*srfr1*) [21], [22], [32]–[34], and indicate that the SAR-like constitutive disease resistance displayed by *cir1* also operates via the EDS1-PAD4 regulatory node.

Given that CIR1 is epistatic to EDS1, we investigated whether CIR1 might regulate EDS1 expression in Arabidopsis. Western blot analysis of the *cir1* mutant revealed that EDS1 protein levels in uninfected plants were higher than in *PR-1:LUC* control plants (Fig. 4A). As *cir1* displays constitutively elevated expression of SA-dependent genes, and *EDS1* gene expression is SA-inducible [11], elevated EDS1 protein levels in *cir1* may simply be a consequence of upregulated *EDS1* transcription in this mutant. However, qPCR analysis revealed no significant difference in steady state levels of the *EDS1* transcript between the *cir1* and *PR-1:LUC* plants (Figure 4B), suggesting that CIR1 may instead function as a negative regulator of EDS1 via a post-transcriptional regulatory mechanism. One possible mechanism for CIR1 action might be via regulation of EDS1 protein accumulation or stability. Analysis of EDS1 protein levels in the *pad4-1* and *cir1 pad4* mutants (Figure 4A) offers limited support to this hypothesis; while EDS1 protein levels were reduced in the *pad4-1* single mutant, in line with previous reports that PAD4 stabilises EDS1 [14], the *cir1 pad4* double mutant displayed similar EDS1 levels to *cir1* plants (Figure 4A). This suggests that the *cir1* mutation may be able to compensate for the lack of PAD4 in the stabilisation of EDS1, and that CIR1 might serve as a negative regulator of EDS1 protein levels *in planta*. Recent studies have indicated that the balance between nuclear and cytoplasmic EDS1 pools is important in EDS1 function in innate immunity [17]. EDS1 protein levels are higher in both nuclei-enriched and nuclei-depleted fractions in the *snc1* mutant [17], and interaction between SNC1 and EDS1 has been detected in both nucleus and cytoplasm [19]. However, the sub-cellular distribution of EDS1 has not been investigated in *cir1*, and so it is unclear whether EDS1 protein levels are elevated in both sub-cellular compartments, or only in one.

Environmental conditions modulate plant-pathogen interactions, with temperature known to play an important role in determining the strength of the host response to pathogen challenge [30], [35]. Higher temperatures within the ambient growth temperature range of the plant have been shown to reduce the effectiveness of both PTI and ETI [35], [36]. The gain-of-resistance mutants *cpr1*, *bon1* and *srfr1* all display temperature modulated growth and defence phenotypes, with dwarfism and increased resistance to biotrophic pathogens observed at 22°C but not 28°C. Similarly, we observed that growth and resistance to *Pst* DC3000 infection are modulated by temperature in *cir1* (Figure 5 & 6). At 18°C an obvious reduction in biomass production in *cir1* was accompanied by enhanced resistance to *Pst* DC3000 relative to *cir1* plants grown at 22°C. In contrast, at 25°C plant size and resistance to *Pst* DC3000 were not significantly different between *cir1* and control plants. While these phenotypes are similar to those reported for *cpr1*, *bon1* and *srfr1*, the temperature at which they occur is lower in *cir1*; while *cpr1*, *bon1* and *srfr1* all exhibit a dwarf phenotype at 22°C, *cir1* displays only a modest reduction in size at this temperature.

The apparently identical temperature sensitivity of *cpr1*, *bon1* and *srfr1* may result from their convergence on the TIR-NB-LRR protein SNC1. Transcript and/or protein levels of SNC1 are elevated in *cpr1*, *srfr1* and *bon1* mutants, indicating that all three proteins act as negative regulators of SNC1 [31], [37], [38]. CPR1 is an F-box protein which interacts *in vivo* with SNC1 [37] suggesting that it regulates SNC1 protein levels via 26S proteasome-mediated degradation. Arabidopsis mutants expressing a constitutively active version of SNC1 (*snc1-1*) display dwarfism, constitutive defence gene expression and increased resistance to biotrophic pathogens [34]. As with *cir1*, *cpr1*, *bon1* and *srfr1*, these phenotypes are temperature dependent, manifesting at 22°C but not at 28°C [31], [36]. Analysis of the progeny from a cross between *bon1* and *snc1-11* (a null allele) has revealed that SNC1 is essential for the dwarfism, constitutive *PR* gene expression and enhanced resistance to biotrophic pathogens displayed by *bon1* at 22°C [31]. Similarly, dwarfism and constitutive *PR-1* gene expression are abolished in the *srfr1 snc1-11* and *cpr1 snc1-11* double mutants [22], [38], suggesting that the auto-immune phenotypes of *cpr1*, *bon1* and *srfr1* at 22°C result largely from de-repression of SNC1.

SNC1 therefore appears to act as a temperature sensor in Arabidopsis to modulate host immunity in response to changes in the environment. Further evidence for this role comes from the *snc1-3* mutant which displays dwarfism and increased resistance to *Pst* DC3000 at both 22°C and 28°C [36]. It has been suggested that a threshold concentration of SNC1 must be reached in the nucleus to trigger immunity, supported by data showing that in wild-type plants SNC1 nuclear content decreases with increasing temperature, but not in the *snc1-3* mutant [36]. Whether the growth and constitutive defence phenotypes of *cir1* also require SNC1 is currently unknown, although we did not observe a statistically significant increase in *SNC1* transcript levels in *cir1* versus *PR-1:LUC* plants at either 18 or 22°C (Figure 7). Analysis of the *cir1 snc1-11* double mutants we are currently generating will address the role of SNC1 in *cir1*. We are also carrying out genetic mapping to identify the *CIR1* gene. First-pass mapping experiments on the F2 progeny of a cross between *cir1* and Ler plants indicated that *cir1* mapped approximately 9.4 cM below nga111 on the lower arm of chromosome 4 [24]. Subsequent linkage analysis to markers within this region has localised *CIR1* to a 46 kb region of chromosome IV, and we are currently analysing candidate genes within this region. Identification of the *CIR1* gene will shed light on the exact biochemical roles played by this negative regulator of EDS1 and PAD4-mediated immunity in Arabidopsis.

## Materials and Methods

### Plant growth conditions

Arabidopsis seeds were stratified for 48 h at 4°C in the dark prior to sowing on either a 1:1 mix of peat (Jiffy Products, Norway) and vermiculite or on half-strength MS agar plates. Plants were grown under a long-day photoperiod (16 h light, 8 h dark) at 22°C (unless otherwise stated) and 55% relative humidity, and cool white fluorescent light of 80–100 µmol m^−2^s^−1^.

### Pathogen assays

All pathogen assays were carried out on four-week old Arabidopsis plants. *Pseudomonas syringae* pv. *tomat*o DC3000 infections were carried out as previously described [39]. *Hyaloperonospora arabidopsidis* Noco2 infections were carried out as described by Parker *et al.* [27], and the extent of plant cell necrosis and development of *H. arabidopsidis* mycelium was examined microscopically 7 dpi by lactophenol trypan blue staining [10].

### Luciferase assays

Total protein was extracted by homogenizing leaf tissue from four-week-old soil grown plants in 1 mL extraction buffer (100 mM sodium phosphate buffer pH 7.2, 5 mM DTT). The samples were centrifuged for 5 min at 12 000×g to pellet cell debris, and 100 µL of the resulting supernatant added to 100 µL of assay buffer (60 mM Tris-HCl pH 8.0, 20 mM MgCl_2_, 20 mM DTT, 2 mM EDTA, 2 mM ATP). Luciferase activity was measured for 20 s following injection with 100 µL of luciferin buffer (60 mM Tris-HCl pH 8.0, 20 mM MgCl_2_, 20 mM DTT, 2 mM EDTA, 1 mM luciferin) in a Luminoskan TL-Plus luminometer (Labsystems, Finland). Luciferase activity was normalised to total protein concentration as determined by Bradford protein assay.

### Generation of *cir1 eds1* and *cir1 pad4* double mutants

Homozygous *cir1* plants were crossed to either *eds1-2* (Ler background) or *pad4-1* (Col-0 background) mutants, and the resulting F1 progeny allowed to self-fertilise. While the marker used to screen for homozygosity for the *cir1* mutation is elevated *PR-1:LUC* activity, both EDS1 and PAD4 are known to be required for *PR-1* expression [13]. Unsurprisingly then, segregation analyses of the F2 progeny revealed a significant deviation from the expected 13:3 ratio of low:high *PR-1:LUC* activity suggesting that the *eds1* and *pad4* mutations were affecting *PR-1::LUC* reporter activity. A two-step process was therefore employed to isolate the double mutants. Homozygous *cir1* plants were identified in the F2 generation by screening for high *PR-1:LUC* activity, comparable to that of the single *cir1* mutant. As expected, genotyping revealed that all were heterozygous for either the *eds1-2* or *pad4-1* mutant allele. The F3 progeny from these plants were then screened by PCR genotyping or RFLP analysis to identify individuals homozygous for the *eds1* or *pad4* mutation. The PCR primers used for genotyping the *EDS1* locus were 5′-GTGGAAACCAAATTTGACATTAG-3′ and 5′-ACACAAGGGTGATGCGAGACA-3′ which generated PCR products of 750 bp (*EDS1*) or 600 bp (*eds1-2*). For PAD4 genotyping, the primers used were 5′-GCGATGCATCAGAAGAG-3′ and 5′-TTAGCCCAAAAGCAAGTATC-3′ which generated a 391 bp PCR product. The *PAD4* amplicon is cleaved by *Bsm*FI to give products of 281 and 110 bp, while the *pad4-1* amplicon is not. As *eds1-2* is in the Ler background, *cir1* was also crossed with Ler to determine whether the mixed Col-0/Ler genetic background affected the penetrance of the *cir1* mutation.

### Quantitative PCR analysis

Total RNA was extracted using Trizol (Invitrogen), treated with DNase and cDNA synthesised from 1 µg of RNA using Superscript III reverse transcriptase (Invitrogen). Quantitative PCR was performed using a RotorGene RG3000A instrument (Corbett Research, Australia). Reactions consisted of 1 µL template cDNA, 5 µL Kapa SYBR FAST Universal 2×qPCR Master Mix (Kapa Biosystems, South Africa), and 200–900 nM of each primer in a final volume of 10 µL. Amplification conditions included an initial step at 95°C for 3 min, followed by 40 cycles of 95°C for 3 s, primer annealing at 60 or 65°C for 20 s and elongation at 72°C for 1 s. Melt curve analysis confirmed that the individual amplified products corresponded to a single, gene-specific cDNA fragment. The relative expression level of each gene of interest was calculated with the RotorGene 6000 series software v1.7 using the two standard curve method, with normalisation to the reference gene *Actin-2* (At3g18780). Details of the primers used and specific qPCR reaction conditions can be found in Table S1.

### Western Blot analyses

Total protein was isolated from leaf tissue as described by Ingle *et al.* [40]. Forty µg of total protein was separated on 12% (w/v) SDS PAGE gels, transferred to nitrocellulose membrane and blocked for 2 h at RT in 1×TBS-T buffer containing 2% (w/v) skim milk powder. Primary EDS1 antibody [13] was diluted 1:400 in 1×TBS-T buffer with 2% (w/v) milk powder, and blots incubated o/n at 4°C. Incubation with primary antibody was followed by 3×10 min washes in 1×TBST, and incubation with secondary antibody (Rabbit IgG HRP, 1:5000 dilution) for 1 h at RT, prior to band detection by chemiluminescence.

### Statistical analyses

Statistical analyses of all data were carried out using Statistica (version 9). *Pst* DC3000 titre data were log-transformed prior to ANOVA to ensure homogeneity of variance and normality of error. Fisher’s LSD post-hoc analysis was used to identify significantly different mean values within an experiment. The raw data obtained from pathogen assays and qPCR experiments that were used in the statistical analyses (and in the generation of Figures 1, 2, 3, 4B, 6 and 7) are provided in Table S2.

## Supporting Information

Figure S1 **Trypan blue-stained leaf tissue of four-week-old plants six days post-infection with** ***Hyaloperonospora arabidopsidis*** **Noco2.**
(PDF)

Table S1 **Primers used in quantitative PCR experiments.**
(PDF)

Table S2 **Data used to generate** **Figures 1** **,** **2** **,** **3** **,** **4B** **,** **6** **and** **7** **.**
(XLSX)
