# Supplementary material for: Increased Resistance to Biotrophic Pathogens in the Arabidopsis Constitutive Induced Resistance 1 Mutant Is EDS1 and PAD4-Dependent and Modulated by Environmental Temperature
Source: PLoS One. 2014 Oct 10;9(10):e109853. doi: 10.1371/journal.pone.0109853 (PMC4193848; doi:10.1371/journal.pone.0109853)
Supplement: Figure S1 — Trypan blue-stained leaf tissue of four-week-old plants six days post-infection with Hyaloperonospora arabidopsidis Noco2. (PDF) [file pone.0109853.s001.pdf]

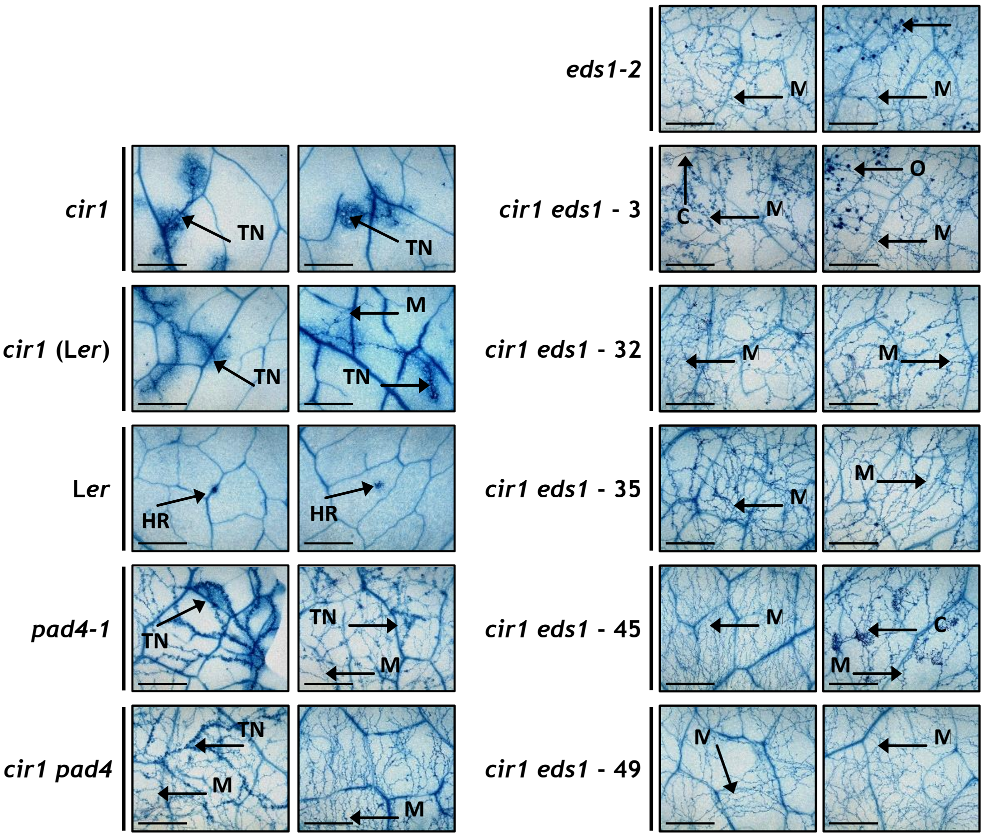

Figure S1. Trypan blue-stained leaf tissue of four-week-old plants six days post-infection with *Hyaloperonospora arabidopsidis* Noco2. Conidiophores (C), hypersensitive response (HR), mycelium (M), oospores (O) or trailing necrosis (TN) are indicated. Scale bars indicate a distance of 0.5 mm.
