# Supplementary material for: Increased Resistance to Biotrophic Pathogens in the Arabidopsis Constitutive Induced Resistance 1 Mutant Is EDS1 and PAD4-Dependent and Modulated by Environmental Temperature
Source: PLoS One. 2014 Oct 10;9(10):e109853. doi: 10.1371/journal.pone.0109853 (PMC4193848; doi:10.1371/journal.pone.0109853)
Supplement: Table S1 — Primers used in quantitative PCR experiments. (PDF) [file pone.0109853.s002.pdf]

**Gene**	**Primer sequence or source and final concentration used in qPCR (nM)**	**Annealing temp (°C)**	**Amplicon size (bp)**
At3g18780 (*Actin-2*)	AGTGGTCGTACAACCGGTATTGT (900)<br>CATGAGGTAATCAGTAAGGTCACGT (300)	60	138
At3g48090 (*EDS1*)	GGTACAGTCGATGGGAAAGAAG (200)<br>GTGCTCACTCCAAGGTCATT (200)	60	87
At2g14160 (*PR-1*)	CATGGGACCTACGCCTACC (900)<br>TTCTTCCCTCGAAAGCTCAA (300)	60	92
At4g16890 (*SNC1*)	GCTCGCCGACTTTACAGACT (200)<br>GGAAGATGATACAACTTATCCCAGA (200)	60	141
At2g31880 (*suppressor of BIR1*)	Arabidopsis pathogen-inducible genes primer library (200)	65	159

Table S1. Primers used in quantitative PCR experiments. Final concentration and annealing temperature used in qPCR and resulting amplicon size are shown. The Arabidopsis pathogen-inducible genes primer library was obtained from Sigma-Aldrich (product number P01000).
